# Individual Level Injection History: A Lack of Association with HIV Incidence in Rural Zimbabwe

**DOI:** 10.1371/journal.pmed.0020037

**Published:** 2005-02-22

**Authors:** Ben A Lopman, Geoff P Garnett, Peter R Mason, Simon Gregson

**Affiliations:** **1**Department of Infectious Disease Epidemiology, Faculty of MedicineImperial College LondonUnited Kingdom; **2**Biomedical Research and Training InstituteHarareZimbabwe; Commission Nationale de Lutte contre le SIDARwanda

## Abstract

**Background:**

It has recently been argued that unsafe medical injections are a major transmission route of HIV infection in the generalised epidemics of sub-Saharan Africa.

**Methods and Findings:**

We have analysed the pattern of injections in relation to HIV incidence in a population cohort in Manicaland in a rural area of Zimbabwe. In Poisson regression models, injections were not found to be associated with HIV in males (rate ratio = 0.33; 95% confidence interval: 0.07 to 1.46) or females (rate ratio = 1.04; 95% confidence interval: 0.59 to 1.85).

**Conclusion:**

It is important that unsafe medical injections can be confidently excluded as a major source of HIV infection. In rural Zimbabwe the evidence is that they can.

## Abstract 2

A debate is raging over whether unsafe medical injections are an important cause of HIV infection in Africa; in rural Zimbabwe evidence suggests they are not

## Introduction

The widely held belief that heterosexual transmission is the driving force behind sub-Saharan Africa's (SSA's) HIV epidemic [1] has recently been questioned [2]. According to Gisselquist and colleagues, investigators have overlooked the importance, and indeed suppressed analysis, of unsafe medical injections as a route of transmission for HIV. Hitherto, assessments of this hypothesis have largely relied on ecological analyses—relating population-level data on unsafe injections to the distribution of HIV prevalence [3,4]. The absence of investigation into the role of unsafe injections, based on the assumed predominance of sexual transmission, has rightly been criticised. However, this criticism trivialises the difficulty of collecting and analysing relevant field data.

Presently, the only published data on the possible contribution of injections to HIV transmission in SSA come from rural Uganda, where Kiwanuka et al. demonstrated no association of injections with HIV incidence [5]. Data from other SSA countries with generalised epidemics where spread has varied in scale and pattern are required to inform this debate.

In this paper, we analyse data from a population cohort in Manicaland in rural Zimbabwe. We describe the determinants of injections in adults and then test the association between injections and incidence of HIV infection.

## Methods

Data were analysed from the baseline (1999/2000) and follow-up (2002/2003) rounds of a cohort of adults in the Manicaland HIV/STD Prevention Study. Eligible men and women aged 15 to 54 were recruited based on an initial household survey [6]. In response to the awakening controversy, a question exploring exposure to injection was added two-thirds of the way through follow-up. Thus, data were available from individuals from four of the 12 study sites. In these sites, 505 males and 1,342 females were interviewed, representing a follow-up of 69.7% of individuals interviewed at baseline. The subset of individuals who were HIV-negative at baseline (*n* = 1,606; 83.6%) was used for all analyses except for the examination of rates of injections stratified by HIV status at baseline.

At follow-up, participants were asked whether they had received an injection or had been pricked by a needle since the baseline interview. A range of health and socio-demographic data were also collected, including self-reported history of sexually transmitted disease (STD) symptoms. Reports on STD symptoms were from the 1-y period before the follow-up interview, and thus did not correspond to the entire 3-y follow-up period. HIV serological testing was performed on dried blood spots using a highly sensitive and specific antibody dipstick assay [7].

Ethical approval for this study was granted by the Medical Research Council of Zimbabwe (MRCZ/A/681) and the Applied and Qualitative Research Ethics Committee, Oxford University, United Kingdom (N97.039). Written informed consent was sought from study participants.

The objective of this analysis was to test the plausibility that injections are an important risk factor for HIV incidence. Having received an injection was modelled as a proximate cause with demographic variables, sexual behaviour, and STDs acting as potential confounders. Determinants of injections were analysed with univariable and multivariable Poisson regression models of incidence rate ratios (RRs). Attributes were retained in age-adjusted multivariable models if the stratum-specific RR differed from one and had a Wald-test *p*-value ≤ 0.1. Using the same strategy, models were then fitted with HIV as the outcome variable.

## Results

Overall, 744 out of 1,847 individuals (40.3%) reported having received an injection or needle prick during the 3-y follow-up period. Females reported more injections than males (RR = 1.93). Rates were not significantly higher for individuals who were HIV positive at baseline (RR = 1.07, *p* = 0.81 for males; RR = 1.13, *p* = 0.28 for females) (Table 1).

**Table 1:**
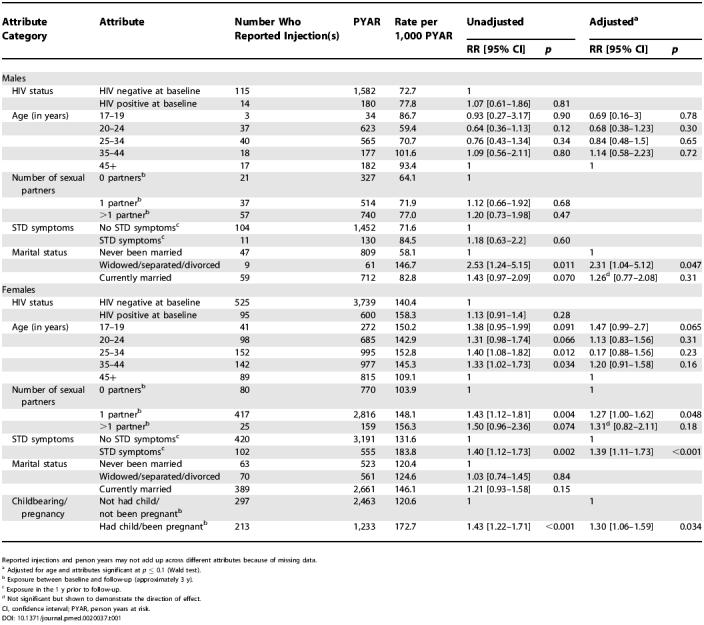
Univariable and Multivariable Poisson Regression Models of Incidence of Injections and Needle Pricks—Presented Separately for Males and Females

Reported injections and person years may not add up across different attributes because of missing data

^a^ Adjusted for age and attributes significant at *p* ≤ 0.1 (Wald test)

^b^ Exposure between baseline and follow-up (approximately 3 y)

^c^ Exposure in the 1 y prior to follow-up

^d^ Not significant but shown to demonstrate the direction of effect

CI, confidence interval; PYAR, person years at risk

Being widowed, separated, or divorced was the only attribute associated with increased rates of injections for males (Table 1). For females, STD symptoms and childbearing/pregnancy were significant in adjusted models (Table 1).

There were 67 HIV seroconversions (48 females and 19 males); 13 (19%) of those seroconverting reported not having had sex in the inter-survey period, and 40 (60%) reported not having had an injection during the period (Table 2).

**Table 2:**
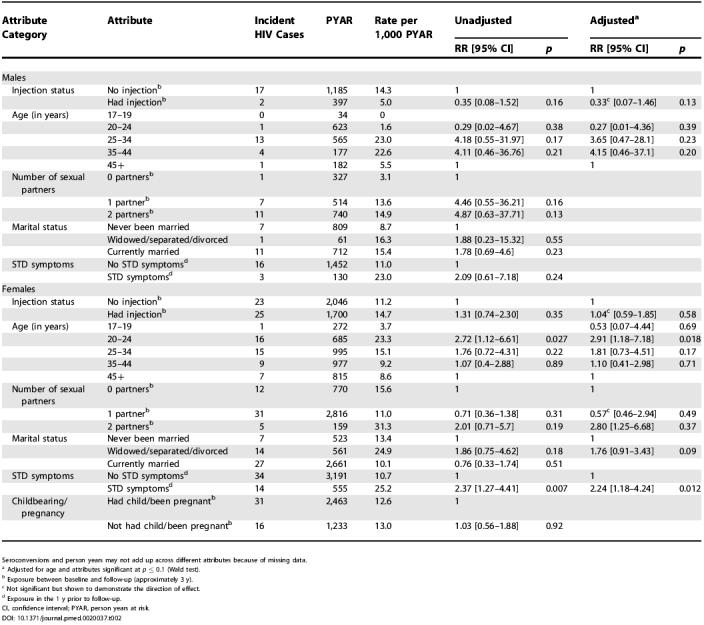
Univariable and Multivariable Poisson Regression Models of HIV Incidence—Presented Separately for Males and Females

Seroconversions and person years may not add up across different attributes because of missing data

^a^ Adjusted for age and attributes significant at *p* ≤ 0.1 (Wald test)

^b^ Exposure between baseline and follow-up (approximately 3 y)

^c^ Not significant but shown to demonstrate the direction of effect

^d^ Exposure in the 1 y prior to follow-up

CI, confidence interval; PYAR, person years at risk

There was no significant association between injections and HIV incidence among either males or females—in either unadjusted or adjusted models. For males, HIV seroconversion rates were elevated amongst 25- to 44-y-olds, sexually active individuals, and those who had suffered STD symptoms, though none of these attributes reached levels of statistical significance (Table 2). For females, having multiple sexual partners, having STD symptoms, and being widowed/separated/divorced were associated with increased HIV incidence. Childbearing/pregnancy, which was strongly associated with injections (see Table 1), had no association with HIV incidence (Table 2).

## Discussion

These data, from a population cohort in rural Zimbabwe, suggest that—at the population level—injections are not a major route of HIV transmission. There was a very slight, non-significant association between injections and HIV amongst females. Could this association achieve significance with a greater sample size or more events? Given the strong association between STD symptoms and both injections and HIV incidence, this is possible, but would likely be a result of residual confounding. In other words, both HIV and injections have a common association with STDs. It has been argued that the association between sexual activity and HIV is confounding between STDs and pregnancy and injections and that it is the injections that are causal. Our analysis does indeed find these associations—but finds STD symptoms the strongest predictor of new HIV infections.

Our measure of injection risk is unambiguous but lacks many dimensions relating to unsafe injections. Presently, we have collected data about the receipt of injections and other needle pricks. Thus, the exposures in these analyses are not restricted to injections received from the health-care sector—the source that Gisselquist et al. originally hypothesized as a major route of transmission [2]. Also, these data reflect only whether an individual had an injection or not—rather than the number of injections received. World Health Organization estimates suggest that the number of injections people receive is not evenly distributed in SSA populations [8].

Thirteen of 67 individuals seroconverting in this study reported no sexual partners in the inter-survey period. Only four of these 13 reported never having had sex. This leads us to suspect that incorrect categorisation of HIV status at baseline in addition to misreporting of sexual behaviour may explain some of these infections: it is possible that a proportion of the nine individuals who reported having had sex in their lifetime but not in the inter-survey period had been recently infected but had not yet seroconverted at the time of the baseline testing [9]. In this scenario, their exposure would have occurred prior to baseline, rather than in the follow-up period. Recall biases may also play a role, given the relatively long follow-up of 3 y [10]. Non-regular partners, especially those from the beginning of the recall period, may have been under-reported. Eliciting accurate reporting of sexual activity is notoriously difficult in Africa and elsewhere, though the use of informal confidential voting interviews has decreased social desirability biases in this cohort [11]. Nonetheless, the cases (*n* = 4) where individuals seroconverted who had reported never having had sex may still be a product of social desirability reporting bias. Clearly, incidence data offer the most explanatory power in elucidating the determinants of the HIV epidemic, but these anomalous cases also highlight the difficulties of collecting time-varying sexual behaviour and serostatus information.

Interestingly, in light of expected HIV-associated disease and care, individuals who were HIV positive at baseline did not have higher rates of injections than individuals who were HIV negative. Injections were found to be highly associated with childbirth/pregnancy. HIV-positive women—and especially those at advanced stages of infection—are known to experience reduced fertility [12]. Therefore, a reduction in use of maternal health services may partially explain why injections were not more common in the HIV-positive population. A more discriminating measure of exposure, including the reason for injection, could help to explain this observation.

Had injections proven to be a risk factor for HIV incidence, further investigations would have been needed to determine the source and types of risky needle pricks. However, no such association was found. Unsafe injections are unacceptable, but this evidence suggests that they do not play a major role in the transmission of HIV in rural Zimbabwe.

**Patient Summary**

**Background**

There is a lot of controversy over whether the spread of HIV in sub-Saharan Africa is predominantly caused by unsafe sexual practices, or whether unsafe medical injections given by health professionals might also have a prominent part to play. A recent paper suggested that unsafe medical injections were important.

**What Did the Authors Do?**

In an ongoing survey in rural Zimbabwe between 1999/2000 and 2002/3 that was trying to assess why some individuals get infected with HIV, the authors asked 505 men and 1,342 women a number of questions. They asked them about their sexual history, whether they had children, and whether they received injections. They tested the adults for HIV at the beginning and end of the study period.

**What Did They Find?**

744 people had had a medical injection and 67 people acquired HIV. There was no evidence overall that injections were linked with an increase in HIV infection. The strongest link with HIV infection was with symptoms of sexually transmitted diseases (in other words, people with these symptoms were more likely to acquire HIV infection).

**What Do These Findings Mean?**

These findings suggest that although it is still possible for an individual to get HIV through unsafe medical injections, overall in this population in Zimbabwe, unsafe injections are not an important cause of HIV infection. Hence policymakers should concentrate more on trying to prevent infection from unsafe sex.

**Where Can I Get More Information?**

Information on safe sex: http://www.thebody.com/safesex.html

World Health Organization Web page on reducing the risk of HIV infection in drug users who inject drugs intravenously: http://www.who.int/hiv/topics/harm/reduction/en/

Fact sheet from the Joint United Nations Programme on HIV/AIDS on HIV/AIDS in Zimbabwe: http://www.unaids.org/html/pub/publications/fact-sheets01/Zimbabwe_en_pdf.htm

## Abbreviations

RR: rate ratio
SSA: sub-Saharan Africa
STD: sexually transmitted disease

## References

[pmed-0020037-b1] Mann J, Tarantola DJM, Netter TW (1992). AIDS in the world: The Global AIDS Policy Coalition.

[pmed-0020037-b2] Gisselquist D, Potterat JJ, Brody S, Vachon F (2003). Let it be sexual: How health care transmission of AIDS in Africa was ignored. Int J STD AIDS.

[pmed-0020037-b3] Gisselquist D, Potterat JJ, Brody S, Minkin SF (2004). Does selected ecological evidence give a true picture of HIV transmission in Africa?. Int J STD AIDS.

[pmed-0020037-b4] Schmid GP, Buve A, Mugyenyi P, Garnett GP, Hayes RJ (2004). Transmission of HIV-1 infection in sub-Saharan Africa and effect of elimination of unsafe injections. Lancet.

[pmed-0020037-b5] Kiwanuka N, Gray RH, Serwadda D, Li X, Sewankambo NK (2004). The incidence of HIV-1 associated with injections and transfusions in a prospective cohort, Rakai, Uganda. AIDS.

[pmed-0020037-b6] Gregson S, Nyamukapa CA, Garnett GP, Mason PR, Zhuwau T (2002). Sexual mixing patterns and sex-differentials in teenage exposure to HIV infection in rural Zimbabwe. Lancet.

[pmed-0020037-b7] Ray CS, Mason PR, Smith H, Rogers L, Tobaiwa O (1997). An evaluation of dipstick-dot immunoassay in the detection of antibodies to HIV-1 and 2 in Zimbabwe. Trop Med Int Health.

[pmed-0020037-b8] Simonsen L, Kane A, Lloyd J, Zaffran M, Kane M (1999). Unsafe injections in the developing world and transmission of bloodborne pathogens: A review. Bull World Health Organ.

[pmed-0020037-b9] Ly TD, Laperche S, Brennan C, Vallari A, Ebel A (2004). Evaluation of the sensitivity and specificity of six HIV combined p24 antigen and antibody assays. J Virol Methods.

[pmed-0020037-b10] Enel C, Lagarde E, Pison G (1994). The evaluation of surveys of sexual behaviour: A study of couples in rural Senegal. Health Transit Rev.

[pmed-0020037-b11] Gregson S, Mushati P, White PJ, Mlilo M, Mundandi C (2004). Informal confidential voting interview methods and temporal changes in reported sexual risk behaviour for HIV transmission in sub-Saharan Africa. Sex Transm Infect.

[pmed-0020037-b12] Zaba B, Gregson S (1998). Measuring the impact of HIV on fertility in Africa. AIDS.

